# Lessons from obesity prevention for the prevention of mental disorders: the primordial prevention approach

**DOI:** 10.1186/s12888-014-0254-3

**Published:** 2014-09-10

**Authors:** Joshua Hayward, Felice N Jacka, Elizabeth Waters, Steven Allender

**Affiliations:** World Health Organization Collaborating Centre for Obesity Prevention, Deakin University, 1 Gheringhap St, Geelong, Victoria 3220 Australia; IMPACT Strategic Research Centre, Deakin University, Barwon Health, Ryrie St, Geelong, Victoria 3220 Australia; Jack Brockhoff Child Health and Wellbeing Program, Melbourne School of Population and Global Health, The University of Melbourne, 5/207 Bouverie St, Carlton, Victoria 3053 Australia

**Keywords:** Obesity prevention, Common mental disorders, Prevention, Intervention design, Complex intervention, Systems

## Abstract

**Background:**

Emerging evidence supports a relationship between risk factors for obesity and the genesis of the common mental disorders, depression and anxiety. This suggests common mental disorders should be considered as a form of non-communicable disease, preventable through the modification of lifestyle behaviours, particularly diet and physical activity.

**Discussion:**

Obesity prevention research since the 1970’s represents a considerable body of knowledge regarding strategies to modify diet and physical activity and so there may be clear lessons from obesity prevention that apply to the prevention of mental disorders. For obesity, as for common mental disorders, adolescence represents a key period of vulnerability. In this paper we briefly discuss relationships between modifiable lifestyle risk factors and mental health, lifestyle risk factor interventions in obesity prevention research, the current state of mental health prevention, and the implications of current applications of systems thinking in obesity prevention research for lifestyle interventions.

**Summary:**

We propose a potential focus for future mental health promotion interventions and emphasise the importance of lessons available from other lifestyle modification intervention programmes.

## Background

The common mental disorders (CMDs) depression and anxiety, are now presenting as major global public health problems. Recent burden of disease studies have attributed as much as 7.4% of global disability adjusted life-years to mental and behavioural disorders, with 2.5% attributable to major depressive disorder (MDD) alone [1]. Although a matter of some contention, the available data suggest an increase in the prevalence of CMDs [2], particularly in young people [3,4]. Importantly, recent evidence from large-scale prospective cohort studies suggest that physical inactivity and unhealthy diet, are related to the genesis of the CMDs [5-8].

The World Health Organization (WHO) framework for the causes of noncommunicable disease (NCD) proposes that physical inactivity and unhealthy diet are among the key modifiable lifestyle risk behaviours that underlie most NCD’s [9]. Recently, there has been an increasing focus on the potential for, and importance of, taking a preventive approach to mental disorders [10] and authors have suggested that programs which also view CMDs as lifestyle-informed NCDs, with population-level lifestyle modification components, may be useful in the prevention of CMDs [11]. In order to elucidate lessons for future CMD prevention, this commentary briefly discusses the relationships between modifiable lifestyle risk factors and CMDs, characteristics of successful preventive approaches to obesity that are of relevance to the prevention of mental disorders, and the role of systems thinking in strengthening lifestyle risk factor interventions.

## Discussion

### Modifiable lifestyle risk factors and common mental disorders

Since 2009 emerging literature has demonstrated the importance of diet quality to the CMDs [11,12]. For example, the most recent meta-analysis in this field has reported a 30% reduction in the risk for depression in those with high adherence to a Mediterranean dietary pattern (RR = 0.68, 95% CI = 0.54–0.86), [13] while a ‘healthy’ diet pattern is also associated with a reduced likelihood of depression (OR: 0.84; 95% CI: 0.76, 0.92) [14]. Although dietary data to date have been largely observational in nature, a recent large-scale European intervention supports the contention that targeting dietary improvement can prevent some cases of CMDs [15]. Knowledge regarding the contribution of physical inactivity to depression risk has also increased; a recent systematic review with 30 included studies concluded that physical activity was negatively associated with a risk of subsequent depression [16]. Individual studies show that as little as 10–29 minutes of daily physical activity may be adequate to reduce relative risk of clinical depression in women (RR = 0.9; 95% CI: 0.84 - 0.96) [7]. A recent review by Sarris et al. [17] examined in detail the evidence for the use of lifestyle modification as a clinical treatment strategy for depression. The report concluded that physical activity, diet, and a range of other lifestyle factors (including mindfulness-based meditation, sleep regulation, social interaction and others) show clear relevance not only for the clinical management of depression, but also for potential population-level mental health promotion. These findings support the contention that diet and physical activity are shared risk factors for many physical and mental disorders and suggest that targeting lifestyle behaviours may be an effective strategy in the prevention of mental disorders.

### Modifiable lifestyle risk factors in obesity prevention

Obesity prevention research now has a 25-plus year history of targeted lifestyle behaviour interventions, beginning in the 1980’s [18]. The “core” of the obesity problem has long been conceptualised as the result of prolonged energy imbalance, driven by the same lifestyle risk factors discussed in relation to CMD above (physical inactivity and unhealthy nutrition). Recently, obesity prevention research has begun to understand the complex nature of the relationships and interdependencies between physical inactivity and unhealthy nutrition, concluding that these behaviours can be resistant to change if targeted in isolation [19]. Recent work in the obesity prevention field therefore provides valuable insights into how CMD prevention may similarly target these lifestyle risk factors and in this paper we examine this in relation to a particularly high risk time of development; that of adolescence.

Waters et al. [20] have reviewed the childhood obesity prevention intervention literature focusing on studies including controlled designs since 1997. The most recent update included 55 obesity interventions that took place between 1993 and 2010. The review located 8 interventions which specifically targeted 13–18 year olds, and reported that all programs targeted physical activity outcomes, and that six of the eight targeted a range of nutrition-related targets. Several studies within this subgroup reported significant increases in the measured lifestyle outcomes, with three studies reporting significant dietary improvements [21-23] and five studies reporting increased indicators of physical activity [21,24-27], however in some interventions these effects were not sustained over time [23].

Some key limitations were noted within these studies. The majority of the evidence reviewed was derived from interventions with short-term funding, based on strategies that optimally require long-term funding support for effect longevity (school-based programs requiring direct funding from investigators, etc.). Accordingly, the overall effectiveness of these interventions was modest (−0.15 (95% CI −0.21, −0.09) BMI-z points, [20]). Leaders in obesity prevention suggest these modest results reflect a failure to anticipate the complexity of drivers of population lifestyle behaviours, the potential plasticity of risk factors when addressed in isolation, and post-intervention effect dropoff. The review noted that the most promise seems to lie with programs which comprehensively target multiple risk factors, coupled with psychosocial support and environmental change [20].

A second theme emerging from obesity prevention is the Community Capacity Building (CCB) approach, an innovative method of developing sustainable skills, resources, and organisational structure, around a shared health promotion goal, within the community itself [28]. This approach addresses complex, interrelated risk factors by using broad community engagement to tailor intervention approaches to the specific set of social and environmental circumstances that exist within that community.

There have been recent interventions drawing on this framework, including the “It’s Your Move” (IYM) project in the Barwon South-West region of Victoria, Australia [29]. The intervention focussed on community engagement to foster flexible intervention strategies across multiple community sectors and organisational levels. The program was deemed to have successfully reduced overweight and obesity in adolescents, and although some nutritional behaviours remained unchanged, the program did observe increases in active transport in the intervention group [29]. Recent analysis suggested that schools which had large increases in readiness for change throughout the intervention demonstrated significant BMI decreases at followup [30].

### Complex interventions in CMD prevention

The importance of taking complex, multi-component approaches to prevention is also increasingly recognised in CMD research. A review by Weare and Nind highlighted several characteristics of successful school-based mental health promotion programmes, finding that universal programmes, which are embedded within the school curriculum and culture, as well as build teacher capacity and knowledge, and involve the wider community, have demonstrated a wide range of benefits to children’s mental health, social, and educational outcomes [31].

An Australian example of this approach was the Gatehouse Project, a group randomised trial employed to address risky health behaviours and improve emotional well-being in secondary school aged children [32]. This approach embedded strategies within the school curriculum to improve students’ emotional management and interpersonal communication skills, while promoting inclusiveness within the classroom. This intervention was successful in reducing risky health behaviours, including substance use and antisocial behaviours. Although this intervention was not successful in directly reducing students’ symptoms of emotional problems [33], the complex strategies employed to achieve improvements in risky health behaviours in this study have been adopted widely around the globe in both high and low income settings. Existing observational data on Australian adolescents supports the contention that using such multi-component, integrated strategies to address lifestyle-related behaviours may result in positive benefits for mental health outcomes in this age group [5,34].

### The systems perspective: new frameworks for working at scale

As understanding of the complexity of lifestyle risk factor interventions has increased, prevention science is observing a gradual shift from individual risk-factor approaches, through multiple risk-factor approaches, community capacity and multi-level approaches, to a recent emphasis on systems thinking as a framework for addressing complexity.

The systems perspective acknowledges not only the existence of the multiple causal factors which drive complex health problems, but highlights their interrelated and “dynamic” associations as an important consideration for any intervention program [35]. Systems thinking has gained some traction is obesity research, being highlighted as the underpinning theory behind population level intervention programmes in Victoria, Australia [36]. The lack of significant inroads into preventing either obesity or CMDs in adolescence supports the need for an alternate approach more able to deal with these complex drivers.

## Summary

A systems perspective, which posits that complex problems lack simple or obvious solutions, shows that prevention efforts must be based in a deeper understanding of the dynamic complexity of modifiable lifestyle risk-factors [19,35]. Current complex, multi-component approaches to CMD prevention have had mixed success but show promise for further development. To capitalise on lessons learned from the obesity prevention sphere, significant collaboration with existing complex population-level lifestyle interventions appears critical.

## Abbreviations

BMI: Body mass index
CCB: Community capacity building
CMD: Common mental disorder
CVD: Cardio vascular disease
DALY: Disability adjusted life year
IYM: It’s your move
MDD: Major depressive disorder
NCD: Non communicable disease
NHANES: National Health and Nutrition Examination Survey
WHO: World Health Organization
